# Urban heat island impacted by fine particles in Nanjing, China

**DOI:** 10.1038/s41598-017-11705-z

**Published:** 2017-09-12

**Authors:** Hao Wu, Tijian Wang, Nicole Riemer, Pulong Chen, Mengmeng Li, Shu Li

**Affiliations:** 1 School of Atmospheric Sciences, CMA-NJU Joint Laboratory for Climate Prediction Studies, Jiangsu Collaborative Innovation Center for Climate Change, Nanjing University, Nanjing, China; 2 Department of Atmospheric Sciences, University of Illinois at Urbana-Champaign, Urbana, USA

## Abstract

Atmospheric aerosol particles (especially particles with aerodynamic diameters equal to or less than 2.5 μm, called PM_2.5_) can affect the surface energy balance and atmospheric heating rates and thus may impact the intensity of urban heat islands. In this paper, the effect of fine particles on the urban heat island intensity in Nanjing was investigated via the analysis of observational data and numerical modelling. The observations showed that higher PM_2.5_ concentrations over the urban area corresponded to lower urban heat island (UHI) intensities, especially during the day. Under heavily polluted conditions, the UHI intensity was reduced by up to 1 K. The numerical simulation results confirmed the weakening of the UHI intensity due to PM_2.5_ via the higher PM_2.5_ concentrations present in the urban region than those in the suburban areas. The effects of the fine particles on the UHI reduction were limited to the lowest 500–1000 m. The daily range of the surface air temperature was also reduced by up to 1.1 K due to the particles’ radiative effects. In summary, PM_2.5_ noticeably impacts UHI intensity, which should be considered in future studies on air pollution and urban climates.

## Introduction

The term urban heat island (UHI) refers to the increased surface temperatures in urban centres compared to those of their suburban surroundings. The phenomenon was first observed in London^1^ and was named by Manley^2^. The UHI phenomenon is a result of the differences in the surface roughnesses, surface albedos, anthropogenic activities and building densities between an urban centre and its suburban surroundings^3–5^, which cause differences in the local boundary layer characteristics and the underlying surface energy balance^6, 7^. For example, during the day, urban centres experience sensible heat convection efficiency reductions because urban areas are aerodynamically smoother than their surrounding suburban regions^8^. As such, the local UHI effect is distinct from large-scale global warming trends^9–11^.

The approaches for quantifying a UHI phenomenon include in situ observations^9, 12, 13^ and remote sensing^4^ as well as numerical modelling^6^, all of which typically compare a climate indicator, such as the surface air temperature, between a location representing an urban environment and another location representing a suburban environment. Previous studies found that UHIs are more pronounced at night than during the day and are larger in the winter than in the summer^14^.

Several studies have recognized the impacts of UHIs on local meteorology and air quality. Differential heating produces mesoscale winds, which help pollutants circulate and move upward, leading to air pollution issues in urban areas^15^. Thus, UHIs also have significant effects on pollutant concentrations, such as those of ozone and particles, due to their feedbacks on boundary layer stability, which decreases the intensity of vertical mixing^16–18^.

Urban centres are also the dominant sources of fine particles, which have important impacts on boundary layer development via their reduction of the amount of solar radiation reaching the earth surface. This reduction affects the surface radiation balance, leading to a decrease in the surface temperature^19, 20^. Numerical models have been used to quantify the radiative effects of aerosol particles. For example, the regional chemistry climate model COSMO-ART has been used in aerosol-climate studies over Europe^21^. This study found a correlation between the aerosol optical depths and changes in regional temperatures. Im *et al*.^22^ concluded that small differences in summer PM_2.5_ levels can cause changes in temperature of 1.5–4.5 K. The chemical speciation of PM_2.5_ matters in this context as it determines whether the particles only scatter light or also absorb light; sulfate aerosols have a strong cooling effect due to their scattering characteristics, which cause the surface temperatures to decrease as sulfate concentrations increase^23–25^. In contrast, the presence of black carbon may reduce the aerosol cooling effect since this particle is the main absorbing component in anthropogenic aerosols^26^. A large black carbon column burden was found to decrease the surface temperature by nearly 2 K in eastern China while warming the atmosphere at the top of the boundary layer^27^.

Although many investigations of fine particles’ radiative forcing and climate effects exist, only a few studies have focused on the impacts of fine particles on UHIs. The radiative forcings of fine particles are different in urban and suburban regions due to the inherently different PM_2.5_ loads^28^, thus causing differences in the surface temperature cooling effects. Our previous study showed that the UHI intensity was weakened by 0.1–0.2 K during the day due to the impacts of fine particles, based on the surface energy balance equation^29^. However, Chang *et al*.^30^ reported that the night-time UHI can be intensified due to increased incoming long-wave radiation, based on remote sensing temperature data from 39 cities in China.

This study uses one-year surface observations of PM_2.5_ concentrations and temperatures as well as numerical modelling to investigate how UHIs could be affected by fine particles, taking Nanjing, a mega city with spreading urbanization located in the Yangtze River Delta of China, as the target city.

## Methodology

### Observational data

The study period spanned from 1st January to 31st December, 2011. The hourly surface temperature data were collected from the Nanjing Meteorological Bureau, and the hourly PM_2.5_ concentration data came from the Nanjing Environmental Monitoring Center. The urban station is Beijige (118°48′37″ E, 31°59′59″N), while Pukou (118°36′7″E, 31°24′5″N) was selected as the suburban station. The Pukou station is situated in western Nanjing and is not influenced by mountains or the ocean. PM_2.5_ was not widely observed in China before 2013. In 2011 in Nanjing, only one urban site produced PM_2.5_ observations, and no suburban sites made PM_2.5_ observations.

However, in 2014, PM_2.5_ observations were available at both the urban site of Xinjiekou (118°47′26″E, 32°2′51″N) and the suburban site of Longtan (119°11′48″E, 32°11′58″N), both of which were used for the model experiments and have collocated surface temperature observation sites. We used these data to analyse the relationship between UHI intensities and the PM_2.5_ concentration differences between the urban and suburban sites.

### Numerical experiments

An online modelling system, the Weather Research and Forecasting (WRF) Model, coupled with Chemistry Version 3.5.1^31, 32^, was used to investigate the influences of fine particles on UHIs. The model system WRF-Chem uses a non-hydrostatic dynamical core and includes emissions of gas phase species and aerosols, gas phase chemical transformations, photolysis, aerosol chemistry and dynamics (including inorganic and organic aerosols) and the removal of gas phases and aerosol species by wet and dry deposition.

The physics scheme in this study was PBL with YSU^33^. The shortwave radiation parameterization was from NASA Goddard^34^, and the longwave parameterization was RRTM^35^. The chemical mechanism was RADM2 (Second Generation Regional Acid Deposition Model; Stockwell *et al*.^36^) with 158 reactions among the gas phases of 36 species. The MADE/SORGAM (Modal Aerosol Dynamics Model for Europe/Secondary Organic Aerosol Model; Ackermann *et al*.^37^; Schell *et al*.^38^) was used for the secondary inorganic and organic aerosols. The emission module included biogenic and anthropogenic contributions. Biogenic emissions were calculated online using Guenther’s scheme^39, 40^. Anthropogenic emissions were supplied from an offline resource, based on the work of MEIC (*Multi-resolution Emission Inventory for China*, http://www.meicmodel.org/dataset-meic.html), which included the species SO_2_, NO_X_, CO, NH_3_, NMVOC, PM_2.5_, PM_10_, BC, OC and CO_2_.

The model was configured with four one-way nested domains using grid resolutions of 81 km, 27 km, 9 km, and 3 km. The initial meteorological fields and boundary conditions were from the NCEP global reanalysis dataset with a 1° × 1° resolution.

**Figure 1:**
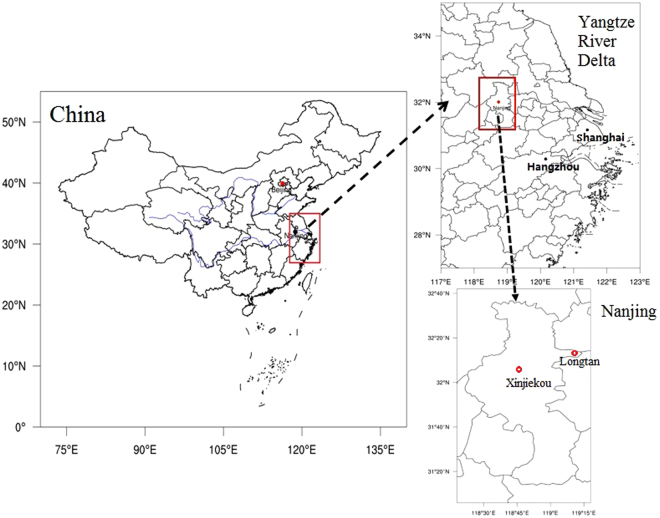
Locations of the urban station (Xinjiekou) and the suburban station (Longtan) in Nanjing, China. The maps were generated using the NCAR Command Language (Version 6.2.0., https://www.ncl.ucar.edu, 2014).

In the numerical experiments, Xinjiekou (118°47′26″E, 32°2′51″N) was chosen as the urban point, as shown in Fig. 1. Xinjiekou is in the central district of Nanjing, close to Beijige, and has no heavy industrial emission sources within 30 km. However, the station is close to major roads with heavy traffic and a large population aggregation. The suburban point is located in Longtan (119°11′48″E, 32°11′58″N), in an eastern district of Nanjing, nearly 40 km away from the city centre of Xinjiekou. Longtan is surrounded by crop fields and inhabited by a small population, similar to Pukou.

To investigate the impact of fine particles on UHI intensities, two sets of numerical experiments were performed for January (winter), April (spring), July (summer) and October (fall), 2011. In the first experiment, the direct and indirect effects of aerosols were disabled. The second experiment included the direct and indirect effects of aerosols. Therefore, the differences of the UHI intensities of the two experiments quantify the effects of PM_2.5_ on the UHI.

In the following sections, the term surface UHI intensity, *T*_UHI_, refers to the difference in the surface temperatures at the urban and suburban stations. The larger *T*_UHI_ is, the more intense the UHI effect. When investigating the PM_2.5_ effect on UHI intensity, we define *∆T*_UHI_ (the difference in *T*_UHI_ between the simulations with and without the PM_2.5_ radiation effect) as the change in the UHI intensity due to the PM_2.5_. A negative value of *∆T*_UHI_ means that the UHI intensity is weakened by the presence of PM_2.5_. We use the variable *∆T* to represent the surface temperature changes including and excluding the PM_2.5_ effect, with negative values of *∆T* representing a lower surface temperature when PM_2.5_ is present. The daily temperature range difference due to PM_2.5_ is denoted by *∆T*_dr_.

### Data availability

The data that support the findings of this study are available from the corresponding author upon reasonable request.

## Results

### Observed PM_2.5_ effects on the UHI

Hourly surface air temperature data at the urban and suburban sites in Nanjing were used to estimate the hourly UHI intensities, *T*_UHI_. To quantify the fine particles’ effects on UHI intensities, we classified the hourly values of the UHI intensities into three groups according to the observed PM_2.5_ concentrations in the urban area, representing light (PM_2.5_ concentrations less than 35 μg/m^3^), medium (PM_2.5_ concentrations greater than or equal to 35 μg/m^3^ and less than or equal to 75 μg/m^3^) and heavy pollution (PM_2.5_ concentrations greater than 75 μg/m^3^).

**Figure 2:**
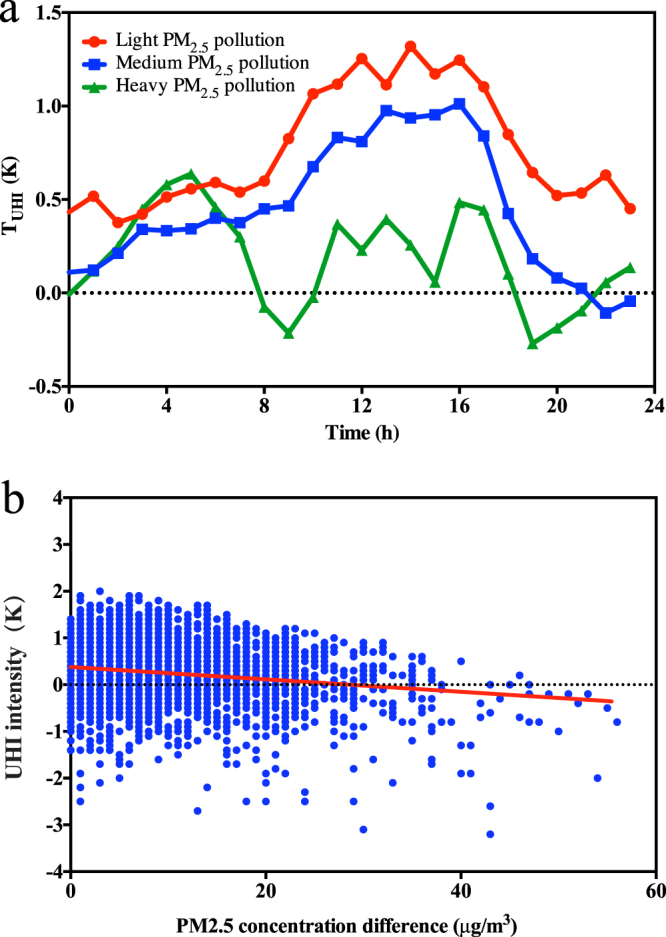
Observed correlations of UHI intensities and PM_2.5_ concentration differences between the urban centre and the suburban station. (**a**) Observed diurnal variations of UHI intensities for different PM_2.5_ levels in Nanjing, 2011. Red, blue and green lines indicate the UHI intensities with light pollution (PM_2.5_ < 35 μg/m^3^), medium pollution (35 μg/m^3^ ≤ PM_2.5_ ≤ 75 μg/m^3^) and heavy pollution (PM_2.5_ > 75 μg/m^3^), respectively. (**b**) Correlation between the observed UHI intensities and PM_2.5_ concentration differences of Nanjing, 2014. Blue dots indicate the UHI intensities and the red line is the linear regression of the UHI intensity and the PM_2.5_ concentration difference. The figure was produced using Prism 6.

Figure 2a compares the diurnal cycles of the *T*_UHI_ of the three pollution levels. The light and medium pollution levels show similar characteristics, with maximum values occurring during the afternoons (1.3 K and 1.1 K for light and medium pollution levels, respectively), and minimum values occurring at night (0.3 K and −0.1 K for light and medium pollution levels, respectively). As the pollution levels increase from light to medium, the UHI intensity decreases for all hours during the day by an average of 0.2 K.

The diurnal cycle of *T*_UHI_ for the heavy pollution case differs from the other two cases, as it does not exhibit a maximum in the afternoon. In contrast, for the heavy pollution case, *T*_UHI_ is larger at night and is approximately 1 K lower than the light and medium pollution cases during the day.

Figure 2b shows the relationship between the UHI intensities and PM_2.5_ concentration differences of the urban and suburban sites (*∆*PM_2.5_) of Nanjing in 2014. Each data point represents an hourly value of a UHI intensity and the corresponding PM_2.5_ concentration difference. The PM_2.5_ levels at the urban site were always equal to or higher than those at the suburban site, with the differences ranging from 0 to 55 μg/m^3^. A negative correlation between the UHI intensity and the PM_2.5_ difference was found; as *∆*PM_2.5_ increased, the UHI intensity tended to decrease.

Chang *et al*.^30^ showed that PM_2.5_ strengthened the UHI at night because it increased longwave radiation. Our results confirm this finding when we compare the light and medium PM_2.5_ loadings to the heavy PM_2.5_ loading. However, when comparing the light and medium PM_2.5_ loading, we find a weakening of the UHI intensity for daytime and nighttime.

### Simulated PM_2.5_ effects on surface temperature

As described in the methodology section, WRF-Chem simulations were carried out to investigate the effects of PM_2.5_ on the surface temperature. Two experiments, one with and one without the direct and indirect aerosol effects, were conducted. The differences of the surface temperatures of the two experiments were used to assess the role of PM_2.5_ on the surface temperature. As fine particles have negative surface radiative forcings, we expect the surface temperatures to become cooler with higher PM_2.5_ loadings^22^.

**Figure 3:**
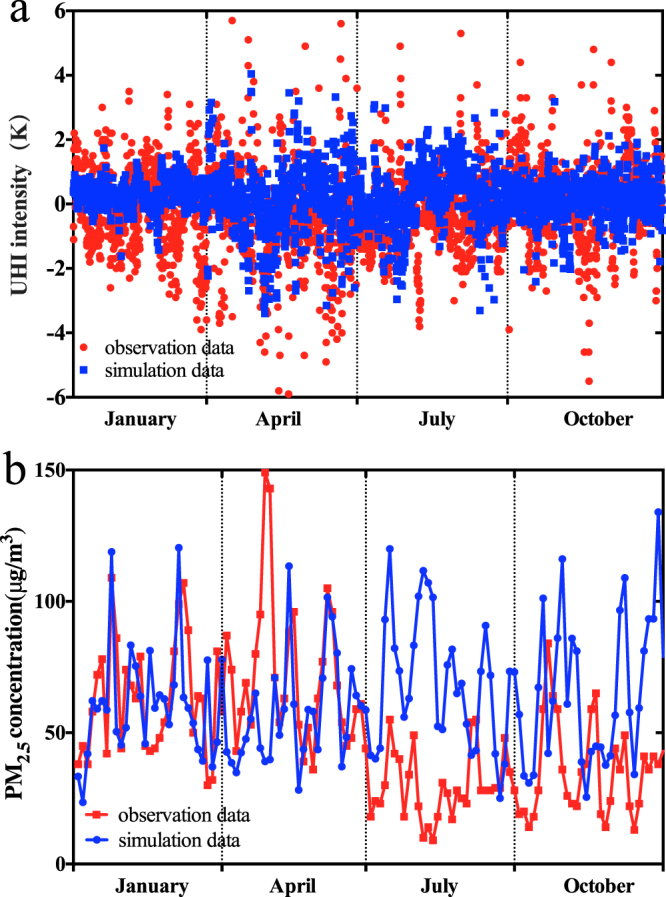
Comparison of observed and simulated UHI intensity and PM_2.5_ concentration in Nanjing, 2011. (**a**) Comparison of hourly observed and simulated UHI intensities between the urban and suburban site. Red dots indicate observational data and blue dots indicate simulation data. (**b**) Comparison of the daily average observed and simulated PM_2.5_ concentration data at the urban site. Red dots indicate the observational data and blue dots indicate the simulation data. The figure was produced using Prism 6.

Figure 3a shows a comparison of the observed and simulated UHI intensities for the station pair Beijige and Pukou for the four months of January, April, July, and October. The monthly averages of the UHI intensities were well captured by the model, as the averages are the same for model and observations (1 K, 0.7 K, 0.2 K and 0.4 K in January, April, July and October, respectively). The model simulation tends to underpredict the range of UHI intensity values on an hourly basis, especially in January. Figure 3b shows a quantitative comparison of the daily observed and simulated PM_2.5_ concentrations at the urban site in Nanjing in 2011. Although the PM_2.5_ levels in July and October are overpredicted by approximately 20 μg/m^3^ and 15 μg/m^3^, the daily variations were well simulated compared to the observations of the four months.

**Figure 4:**
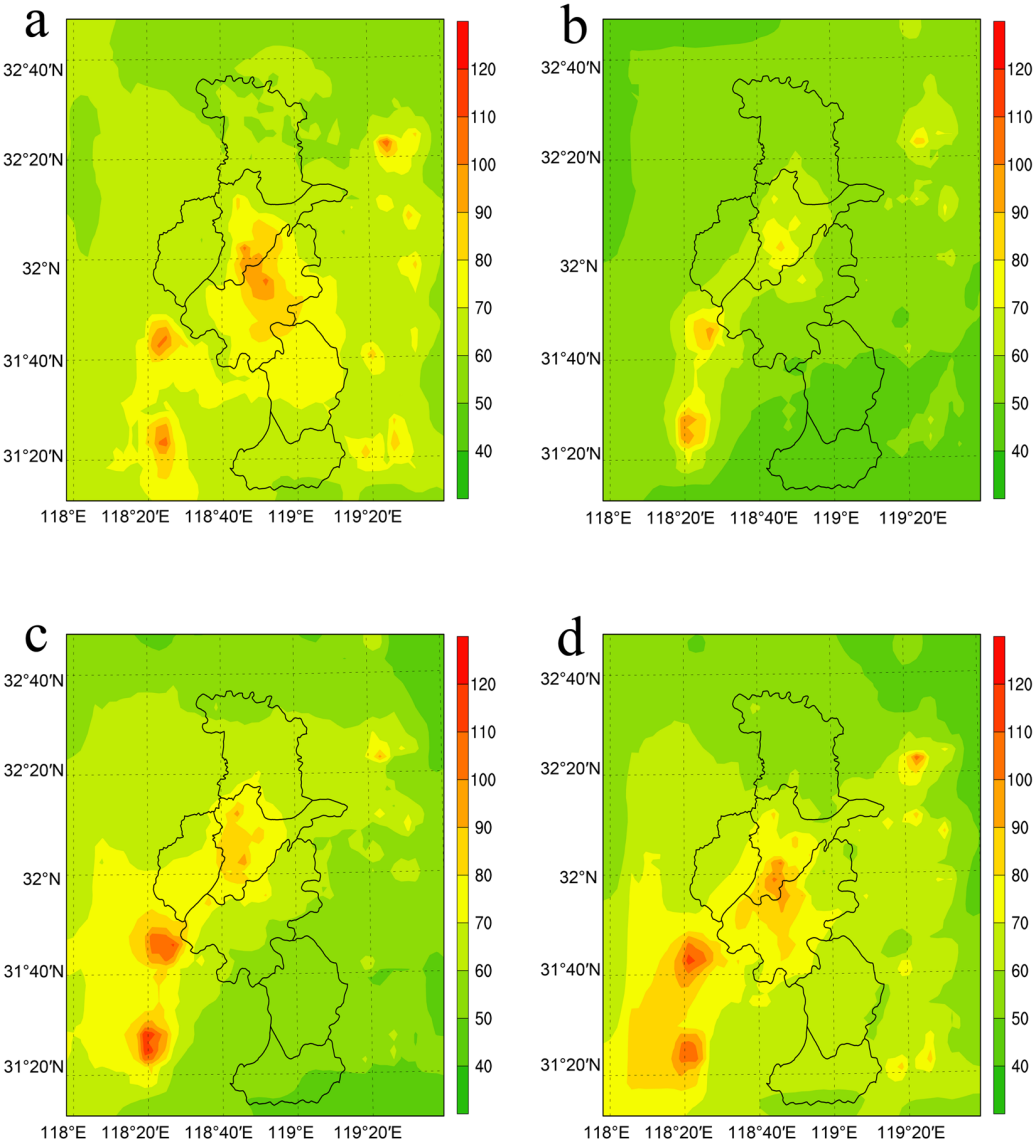
Monthly averaged simulated surface PM_2.5_ concentrations (μg/m^3^) for Nanjing. (**a**) January; (**b**) April; (**c**) July; (**d**) October, 2011. The maps were generated using NCAR Command Language (Version 6.2.0., https://www.ncl.ucar.edu, 2014).

Figure 4 shows the horizontal distribution of the monthly averaged PM_2.5_ concentrations over Nanjing from the output of the lowest model layer. In the urban centre, the average PM_2.5_ concentration in 2011 was observed to be 40–50 μg/m^3^ in the summer (June, July, August) and 80–90 μg/m^3^ in the winter (December, January, February). In the suburban region, the average concentration was observed to be 30–40 μg/m^3^ in the summer and 60–80 μg/m^3^ in the winter, according to short-term observations^29^. The lower PM_2.5_ concentrations in the summer can be explained as being the result of the increased amount of precipitation and better diffusion conditions induced by more instable atmosphere during the summer, resulting in increased surface PM_2.5_ removal by wet deposition and vertical diffusion. Figure 4 shows that the simulations capture the seasonality of the PM_2.5_ concentration. The simulated PM_2.5_ concentration is highest in January (110–80 μg/m^3^ in the urban centre) and lowest in July (100–70 μg/m^3^ in the urban centre). Two other regions of high PM_2.5_ concentrations are apparent near southwestern Nanjing: the cities of Maanshan and Wuhu. The high PM_2.5_ concentrations of these two cities affect the southern suburban areas of Nanjing and do not impact the suburban stations of our numerical experiments.

**Figure 5:**
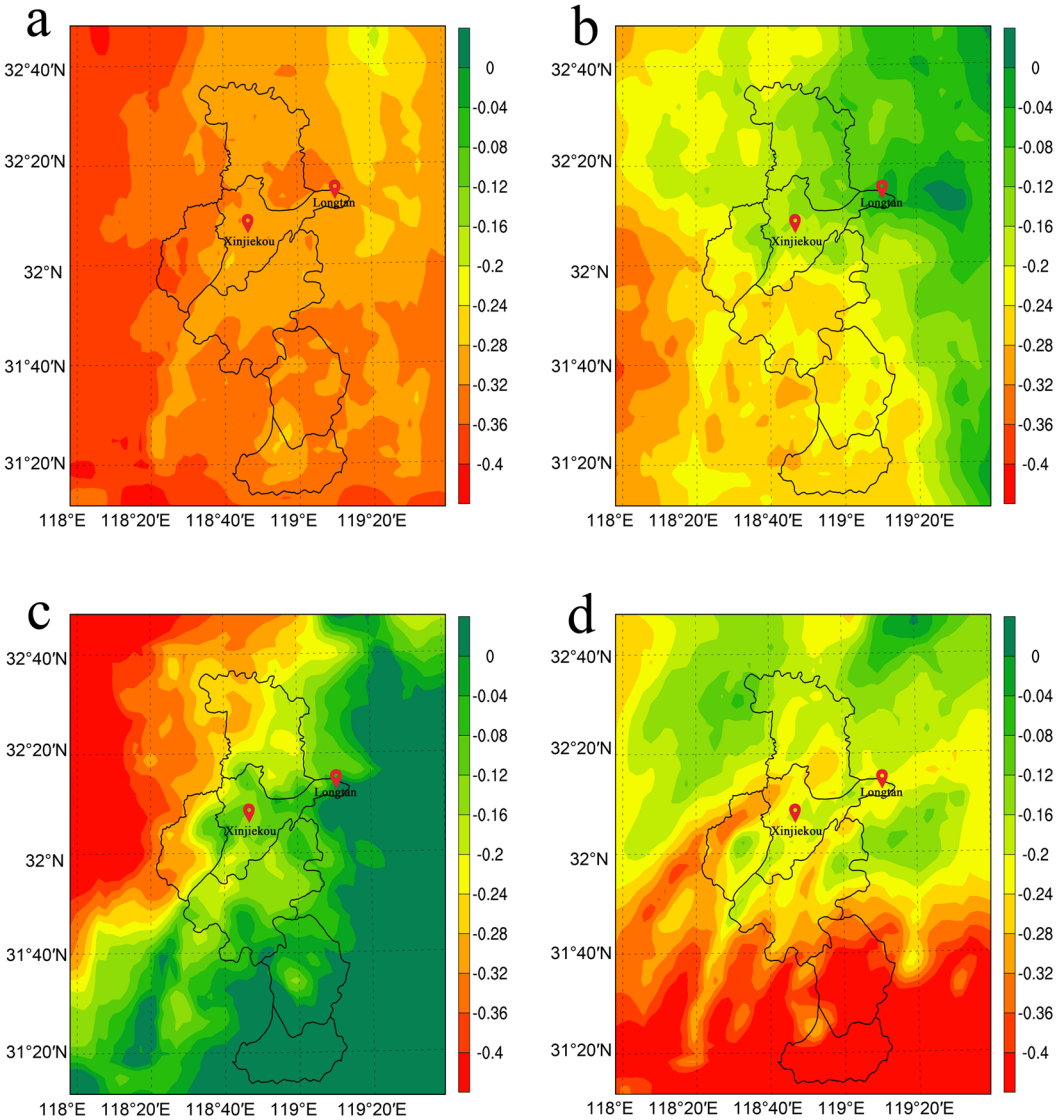
Monthly averaged surface temperature change *∆T* (K) at a 2-m height due to PM_2.5_ in (**a**) January; (**b**) April; (**c**) July; and (**d**) October, 2011. The maps were generated using NCAR Command Language (Version 6.2.0., https://www.ncl.ucar.edu, 2014).

Figure 5 shows the horizontal distribution of the surface temperature differences caused by PM_2.5_. Owing to the seasonal variation of the PM_2.5_ concentration, the surface temperature change (*∆T*) over the area is generally highest in January and lowest in July. However, to determine the impact of PM_2.5_ on the UHI (quantified via *∆T*_UHI_), we need to compare the *∆T* of the urban area to the *∆T* of the suburban area, as a simple correlation with the PM_2.5_ map cannot be expected. Aerosols both scatter and absorb solar radiation, thus cooling the surface. However, the simulated surface temperature is still dependent on other factors; when the aerosols cool the surface temperature, the atmosphere becomes more stable, which changes the moisture transport and results in cloud and precipitation feedbacks, which affect temperature again. Due to these complex feedbacks, the horizontal distribution of the surface temperature response is not always similar to the horizontal distribution of the PM_2.5_ differences. However, the surface temperature gradient around the PM_2.5_ source can be found in Figs 4 and 5.

### Simulated PM_2.5_ effects on the surface UHI

According to the observational results discussed in Section 3.1, the UHI intensity is weakened during the day as the PM_2.5_ concentrations increase. From the simulations, we learn that the surface temperature cooling is indeed inhomogeneous across the domain (Fig. 5). Higher reductions of the surface temperature, as large as 0.3 K, were found in the urban centre, especially in July, while the reduction of the surface temperature was only 0.2 K in the suburban region.

**Figure 6:**
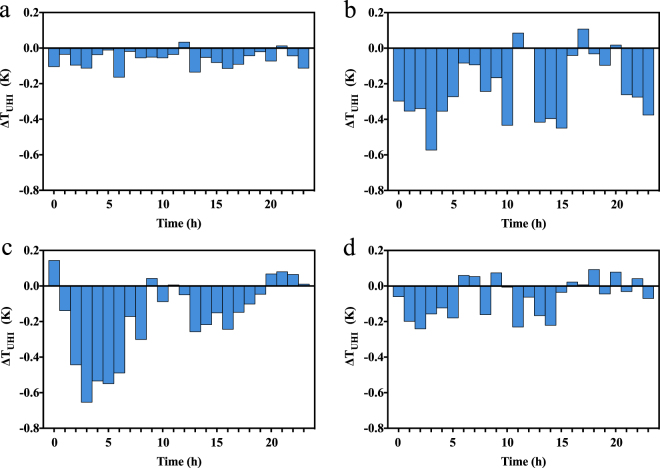
Monthly averages of the diurnal cycles of the simulated changes in UHI intensities due to PM_2.5_ (*∆T*_UHI_ **)** for (**a**) January, (**b**) April, (**c**) July, (**d**) October, 2011. The shaded bars indicate the values of *∆T*_UHI_. The figure was produced using Prism 6.

Next, we quantified the diurnal variations of the simulated changes in the UHI intensities due to the presence of PM_2.5_, *∆T*_UHI_. Negative values of *∆T*_UHI_ correspond to weakened UHI intensities caused by the presence of PM_2.5_. Figure 6 shows the diurnal cycle of the changes in the UHI intensities caused by those of PM_2.5_ over the four different months. During the day, the surface temperature was reduced due to decreased solar radiation caused by the scattering and absorbing aerosols in both the urban and the suburban region. However, since the PM_2.5_ loading of the urban region is consistently larger than that in the suburban region, the temperature decrease was larger in the urban region, which explains the decreased UHI intensity when PM_2.5_ was included in the simulations. While *∆T*_UHI_ is positive at a few points, the magnitudes of these points are small. Thus, the strengthening of the night-time UHI intensity caused by heavy pollution conditions seen in the observational data (Fig. 2) is not found in the simulation results (Fig. 6) because we do not separate the low, middle and high pollution days when calculating the monthly average diurnal UHI intensity changes.

Figure 6 also shows that the magnitude of the *∆T*_UHI_ was larger in April and July than in January and October. Table 1 shows the monthly averaged *T*_UHI_ values with and without the PM_2.5_ effects and the resulting *∆T*_UHI_. The seasonal variations of *∆T*_UHI_ depend on both the available solar radiation and the differences of the PM_2.5_ levels of the urban centre and the suburban area. Without the PM_2.5_ effect, *T*_UHI_ mainly depends on solar radiation, which varies according to the seasons. As a result, *T*_UHI_ was highest in July and lowest in January. However, PM_2.5_ obviously weakened the UHI intensity, and the differences of the PM_2.5_ levels of the urban centre and the suburban area are larger in July (18.51 μg/m^3^) than in January (15.86 μg/m^3^). When including PM_2.5_ in the simulations, *T*_UHI_ reached a lowest value of nearly 0.15 K in July and a highest value at nearly 0.3 K in January. In general, PM_2.5_ contributed to the *∆T*_UHI_ the most, with −0.27 K in July, and the least, with −0.08 K, in January, due to the seasonal variations of the PM_2.5_ concentrations and solar radiation.

**Table 1:** Monthly averaged values of *T*_UHI_ and *∆T*_UHI_ at 2-m heights affected by PM_2.5_.

	January	April	July	October
*T*_*UHI*_ without the PM_2.5_ effect (K)	0.37	0.40	0.42	0.36
*T*_*UHI*_ with the PM_2.5_ effect (K)	0.29	0.19	0.15	0.18
***∆T***_***UHI***_ **(K)**	**−0**.**08**	**−0**.**21**	**−0**.**27**	**−0**.**18**

### Simulated PM_2.5_ effect on the UHI profile

**Figure 7:**
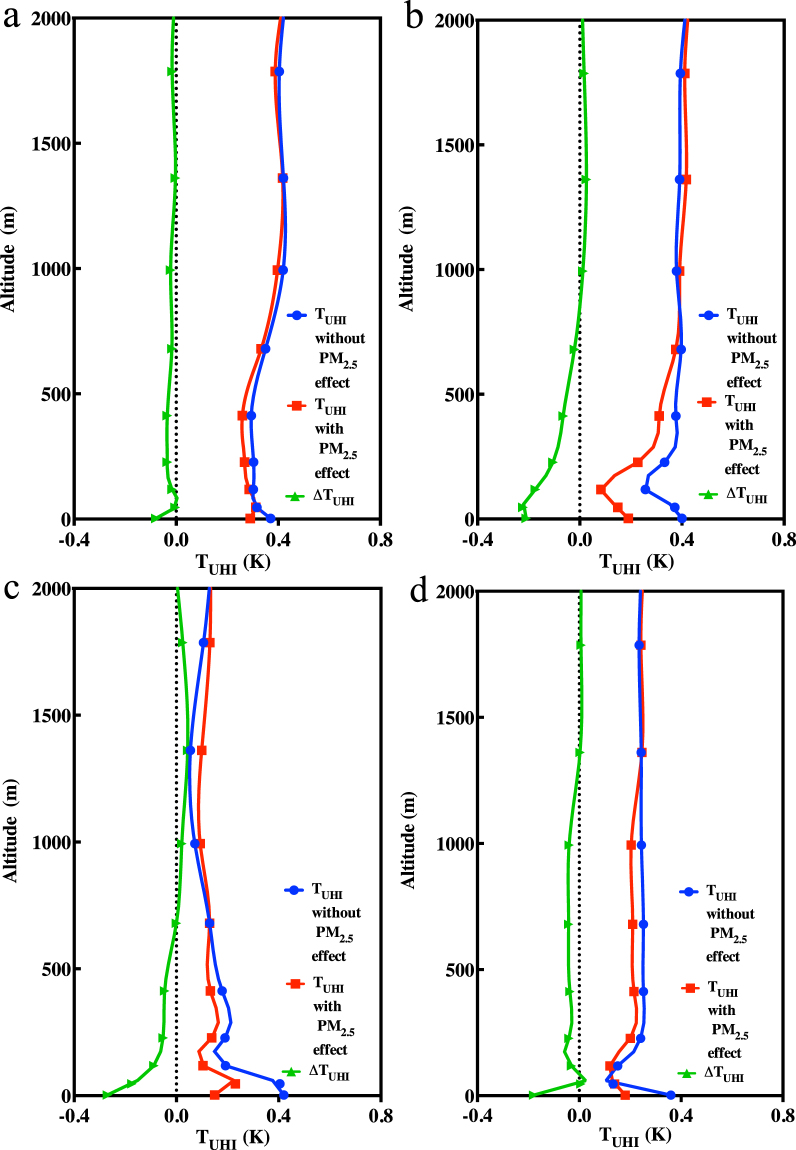
Monthly average *T*_UHI_ profile from 2 m to 2000 m in (**a**) January; (**b**) April; (**c**) July; (**d**) October. Blue, red and green lines indicate the UHI intensities without the PM_2.5_ radiation forcing, with the PM_2.5_ radiation forcing and the differences of the former two cases at different altitudes.

While we have discussed changes in the UHI intensities near the surface caused by PM_2.5_, the changes in temperature due to PM_2.5_ also exist in higher layers. Figure 7 shows the vertical profiles of the UHI intensities with and without including the effects of PM_2.5_, as well as the change due to PM_2.5_. In the four months studied, the *T*_UHI_ values have similar vertical profiles, first declining from the surface up to 100 m, then increasing to a height of approximately 1000 m and remaining constant between 1000 m and 2000 m. This finding shows that the UHI intensity changes can be observed at well over 500 m^41^. The *∆T*_UHI_ profile shows that PM_2.5_ reduces the UHI intensity at lower altitudes, usually below 500–1000 m. For higher altitudes, the *∆T*_UHI_ values approach zero in January and October. In April (Fig. 7b) and July (Fig. 7c), the *∆T*_UHI_ values are larger than zero when the altitude is over 1000 m and decrease to zero at 2000 m. The UHI intensity was most weakened by PM_2.5_ near the surface, but the weakening effect decreases with height. Therefore, the ∆*T*_UHI_ shown in Fig. 7b,c approach 0 K in the 600–700 m layer, with negative values below this height. The small positive values of ∆*T*_UHI_ shown in Fig. 7b,c above 600–700 m mean that the UHI intensity was intensified by as little as 0.03 K, which may be the result of the aerosol warming effect, which is caused by the absorption of solar radiation in the upper levels of the boundary layer. This phenomenon is dependent on the chemical composition and vertical distribution of PM_2.5_, which should be addressed in more detail in a future study. This seasonal variation occurs because PM_2.5_ is mainly limited to the boundary layer, and the top of the boundary layer is higher in the spring and summer than in the fall and winter.

### PM_2.5_ effect on the daily temperature range

The change of the daily temperature range (referred as *∆T*_dr_ in following text) is a useful metric to demonstrate the effects of PM_2.5_ in the urban centre and over the suburban area^42^. Table 2 shows the changes of the daily temperature ranges caused by PM_2.5_ over the four months. In each month, PM_2.5_ reduced the daily temperature range. Considering PM_2.5_’s cooling effect during the day and its weak warming effect at night, the reduction in the daily temperature range is caused by both maximum temperature decreases and the minimum temperature increases, which is true in both the urban and the suburban regions. The magnitude of the *∆T*_dr_ affected by PM_2.5_ is larger in the urban centre than in the suburban region.

**Table 2:** Monthly variations of temperature daily ranges in 2011, Nanjing (K).

	With PM_2.5_ effect	January	April	July		Octo ber
Urban daily temperature range	Yes	6.95	10.95	8.71		7.98
Urban daily temperature range	No	7.04	11.47	9.81		8.05
**Urban** ***∆T***_**dr**_	**—**	**−0**.**09**	**−0**.**52**	**−1**.**1**		**−0**.**07**
Suburban daily temperature range	Yes	6.94	10.68	8.45		8.04
Suburban daily temperature range	No	6.98	10.77	9.33		8.35
**Suburban** ***∆T***_**dr**_	**—**	**−0**.**04**	**−0**.**09**	**−0**.**88**		**−0**.**31**

## Conclusions

Our study reveals that fine particles weakened the UHI intensities during the day using observational analysis and numerical modelling. Based on observations, the UHI intensity can be reduced by up to 1 K under heavily polluted conditions. According to our simulations, PM_2.5_ reduced the surface radiation and the surface temperature^22, 27^ by up to 0.3 K in the urban centre of Nanjing. The simulated PM_2.5_ concentrations were higher in the urban centre than those in the suburban area for all four studied months, thus affecting the UHI intensities. The simulated PM_2.5_ concentration differences between the urban and suburban sites were, on average, 15.86 μg/m^3^, 20.42 μg/m^3^, 18.51 μg/m^3^, 16.73 μg/m^3^ in January, April, July and October, respectively. The seasonal variations of the PM_2.5_ differences over the four months are consistent with those of ∆*T*_UHI_, where the UHI intensity is weakened less in January and October but more in April and July. The day-time UHI intensity reduction was strongest in July and weakest in January as both the PM_2.5_ concentration differences between the urban and suburban areas and the incoming solar radiation varied across different seasons. The reduction mainly occurred at altitudes below 500–1000 m. The response of the UHI intensity to the PM_2.5_ at night depended on the PM_2.5_ load. The night-time UHI intensity was weakened when the pollution levels were low to medium but was strengthened when the pollution levels were high.

PM_2.5_ also reduced the daily temperature ranges, according to the simulations. The daily temperature range values in the urban centre were greater than those in the suburban area for all four months, as was the reduction caused by PM_2.5_.

The UHI intensity and daily temperature range are significant reference signals of the urban climate. We showed that PM_2.5_ pollution reduces urban warming during the day but can intensify the warming at night when the pollution levels are high. Thus, pollution can mask the UHI effects, and pollution levels need to be considered when comparing the UHI intensities of different cities. In a future study, we will further investigate the impact of the PM_2.5_ compositions, as the scattering and absorbing aerosols cause different radiative forcings.
